# Degradation of Aα and Bβ chains from bovine fibrinogen by serine proteases of the Amazonian scorpion *Brotheas amazonicus*

**DOI:** 10.1186/1753-6561-8-S4-P12

**Published:** 2014-10-01

**Authors:** Andre Higa, Maria das Dores Noronha, Jorge Luis López-Lozano

**Affiliations:** 1 Universidade do Estado do Amazonas, Manaus, Amazonas, Brazil; 2 Fundação de Medicina Tropical Doutor Heitor Vieira Dourado, Manaus, Brazil

## Background

Proteolytic enzymes within venoms from different scorpion species belong to serine and metalloproteases class, and some isoforms of these enzymes show proteolytic activity over Aα and Bβ subunits of fibrinogen [1-3]. *Brotheas amazonicus *is a non-lethal amazonian scorpion, belonging to a different group from *Tityus *genus, and there are scarce studies about its venom in literature [4]. Enzymes acting over fibrinogen can lead to the creation of more efficient antithrombotic drugs, and the low lethality of *B. amazonicus *venom is a potencial factor for developing a better drug.

## Material and methods

Venon was early incubated with metallo or serine proteases inhibitors (PMSF and EDTA), and proteolytic activity was evaluated by zymogram in SDS-PAGE using as substrate bovine fibrinogen. After electrophoresis, gel was rinsed with 2.5% Triton X-100. Gel was incubated in humid chamber for 24 hours at 37ºC, in 0.1M Glycine pH 8.3 solution. Dye was performed using 0.02% Comassie Blue R-250 solution. Proteolytic activity of *B. amazonicus *venom over bovine fibrinogen was tested, by mixing 200 µL of fibrinogen solution (2µg/µL) in 0.01M PBS pH 7.4 with 50 µg of *B. amazonicus *venom, for 12 hours at 37ºC. After this process, no clots were observed, so 5µg of *Bothrops atrox *venom [5] was incorporated to the system, but also no clotting was observed, suggesting *B. amazonicus *venom had activity over fibrinogen, but with no clotting formation. Changes induced by *B. amazonicus *venom on bovine fibrinogen were evaluated by 12% SDS-PAGE electrophoresis stained with silver nitrate. Inhibition efficacy of fibrinogenolytic activity of *B. amazonicus *venom by anti-scorpionic serum was tested, by adding different concentrations of anti-venom in 200µL of bovine fibrinogen (2µg/µL) plus 5µg of *B. amazonicus *venom. This system was incubated for 24 hours at 37ºC, and after this process clotting induction by 5µg of *B. atrox *venom was performed.

## Results and conclusion

Proteolytic activity of *B. amazonicus *venom over bovine fibrinogen was only inhibited by PMSF - specific inhibitor for serine proteases. *B. amazonicus *venom degraded bovine fibrinogen without fibrin clots formation, confirmed by clots absence when *B. atrox *venom was incorporated to the system. In SDS-PAGE electrophoresis of degraded fibrinogen, it was possible to detect that *B. amazonicus *venom degraded Aα and Bβ subunits of fibrinogen, and anti-scorpionic serum specific for *Tityus *species shows great neutralizing efficacy when 1:1 proportion, suggesting that *B. amazonicus *toxins show similar antigenic properties of serine proteases from *Tityus *genus venom. Results suggest a serine protease with bovine fibrinogen affinity, able to degrade different regions from this molecule unlike thronbin and with a high similarity of proteases from *Tityus *sp. Such characteristics, plus the fact that this venom has a low toxicity, make these proteases inside *B. amazonicus *venom as candidates for antithrombotic drugs or even vaccines against scorpionic accidents.
